# Effective coverage as a new approach to health system performance assessment: a scoping review

**DOI:** 10.1186/s12913-018-3692-7

**Published:** 2018-11-23

**Authors:** Ali Jannati, Vahideh Sadeghi, Ali Imani, Mohammad Saadati

**Affiliations:** 1 Health Services Management, Iranian Center of Excellence in Health Management, Health Services Management Department, School of Management and Medical Informatics, Tabriz University of Medical Sciences, Tabriz, Iran; 2 Health Services Management, Health Services Management Department, School of Management and Medical Informatics, Tabriz University of Medical Sciences, University Street, next to Shahid Madani hospital, Tabriz, 5165665811 Iran; 3 Pharmacoeconomics and Pharmaceutical Management, Tabriz Health Services Management Research center, Health Economics Department, School of Management and Medical Informatics, Tabriz University of Medical Sciences, Tabriz, Iran; 4 Health Services Management, Road Traffic Injury Research Center, Tabriz University of Medical Sciences, Tabriz, Iran

**Keywords:** Effective coverage, Health system, Performance assessment, Scoping review

## Abstract

**Background:**

Delivering interventions is the main task of health systems whose accurate measurement is an essential input into tracking performance. Recently, the concept of effective coverage was introduced by World Health Organization to incorporate into health system performance assessment. The aim of present scoping review was mapping the key elements and steps of effective coverage assessment in practical efforts including kinds of interventions, criteria for selecting them and the need, use and quality estimation approaches and strategies of each intervention.

**Methods:**

We conducted a scoping review of health system/program assessments which assessed effective coverage till May 2017. Seven databases were systematically searched with no time and language restriction through applying combined keyword of “effective coverage”.

**Results:**

Eighteen studies contributed findings on monitoring effective coverage of health interventions and they all were included in the review. Only 4 contributed findings on health system and the others were related to specific intervention(s) assessment. The interventions monitored by effective coverage were mainly in child health, prenatal and antenatal care and delivery, and chronic conditions areas. Potential impact on the burden of disease, leading causes of mortality and morbidity, and high occurrence and prevalence rate were among the main intervention selection criteria. Availability of data was the critical prerequisite, especially, in all of the studies applied ex post approach in estimating effective coverage. Estimation based on a norm, self- reporting from surveys and biomarkers were the main strategies and methods of need, utilization and quality measurement, respectively.

**Conclusions:**

More studies are needed to contribute to the ongoing improvement in the development of effective coverage concept and increasing practical efforts, especially through defining prospective approaches and strategies into estimation of composite measures based on the effective coverage framework. Also, further attention needs to be paid to quality measures of effective coverage in a manner that better conceptualizes and measures the connection between coverage rates and interventions’ effectiveness. At the administrative system level, more innovation is needed to develop data systems in order to enhance capacity of routine health service information.

**Electronic supplementary material:**

The online version of this article (10.1186/s12913-018-3692-7) contains supplementary material, which is available to authorized users.

## Background

### Health system performance assessment

Delivering prevention and care interventions through a range of health services to people requiring them is the main task of health systems. Further, accurate measurement of such an effort is, therefore, an essential input into tracking performance, decision-making processes and good policy formulation. One measure to determine how well a program is performing is the coverage level it achieves [1, 2]. Coverage data are also needed to monitor the effectiveness of strategies required to reach them [3].

The World Health Organization (WHO) report in 2000 distinguishes between the final goals of health systems —namely health, responsiveness, and fair (financial) contribution and intermediate goals. In addition, it emphasizes that intermediate goals play an instrumental role in achieving the final ones. Expanding performance measurement to intermediate goals, therefore, has been indicated as a reliable way to directly assess the links between health system performance and policy formulation at local, regional, and national levels [4, 5]. So far, many intermediate goals such as access, effectiveness, efficiency, acceptability, and continuity have been suggested and applied to the performance measurement of health systems [5]. Traditionally, monitoring improvements in health system and performance assessment efforts, especially in public health, focuses on such indicators [6]. However, it has been found that increases and improvements in availability, access and use of health services, especially among poor populations, do not always translate into the improvements in health gain [7]. Although the mentioned indicators are important, they do not reflect the effectiveness or quality of the care provided by the health system or the extent to which the key interventions are implemented as intended [6].

Over recent years, there has been some debate about how coverage should be defined, what an acceptable level of coverage should be, how it might vary with the intervention type, and how it can be measured [8]. In 2003, the concept of effective coverage was introduced and its measurement was suggested by WHO to incorporate into health system performance assessment [9].

### Effective coverage

As mentioned above, regarding the default of crude coverage as a proxy indicator for assessing the output of health system and its mere focus on concepts such as availability, access, and utilization as well as lack of its relation to health gain, combined and integrated concept of effective coverage emerged, was introduced and then entered the public health discourse based on preliminary efforts of Shengelia and colleagues in early of 2000 [10]. In their work, effective coverage combines three widely used components of need, utilization, and quality of healthcare interventions, defined formally as the fraction of potential health gain that can be delivered through an intervention by the health system, which is actually delivered [1, 10]. Crude coverage simply takes the fraction of those who use an intervention into account without the quality component (gain in health) while the effective coverage adjusts this concept for the quality or effectiveness of the intervention [7, 11]. Effective coverage (EC) is estimated through using the following formula:

$$ \mathrm{EC}=\mathrm{U}/{\mathrm{N}}^{\ast }\ \mathrm{Q} $$

where N is the population in need of an intervention, U is the utilization/use of the intervention among the population in need, and Q is the quality of the intervention defined as “the ratio of health gain delivered through an intervention relative to the maximum possible health gain given the ideal quality” [10].

The traditional coverage of an intervention (crude coverage) is obtained from U/N ratio, which shows the people who use an intervention among those who need it. Here, there is no linkage between the use of the intervention and the related health gain. We don’t know whether this use results in expected health gain (what it is defined as quality^1^) or not. If U/N equals 1, it means that the people who need an intervention use it. This value (1 or %100) shows the given intervention’s crude coverage. To get the effective coverage, this ratio should be adjusted to quality or health gain value, which is between 0 and 1 where 0 is no health gain and 1 is the maximum gain possible for that intervention. By multiplying the crude coverage ratio and quality, effective coverage of the intervention will be achieved. If it is 50%, then only half the potential health gain from the intervention is expected to be delivered. For example, if measles vaccination can reduce measles incidence by 90%, but, due to cold chain failures and other problems, a program only reduces incidence by 45%, then, the quality or effectiveness adjustment to crude coverage would be 50% [1].

In fact, in this section, we shift attention to the *“actual delivery*” of interventions to those who need them [12].

Lack of data at coverage levels and proper tools to assess performance prevents the identification of priorities for action and leads to insufficient measurements of key health system functions and outputs [12, 13]. Despite the emphasis, little is known about the position of the countries with respect to providing effective health services [14]. It is now a priority for public health surveillance, therefore, to provide a comprehensive and systematic approach to measuring coverage of health interventions according to new metrics such as effective coverage [15, 16] in order to know how well the programs serve those who need them [17]. Effective coverage, therefore, could be a decent intermediate goal that would meet the aforementioned criteria and can be used to trace the degree to which the health system carries out critical activities that have an impact on people’s health [5].

Following the introduction of the concept of effective coverage and its entry into the field of health literature, several efforts have been conducted to develop this concept and apply methodologies for its measurement at national, regional and global levels. We did a scoping review aiming to gather these efforts to identify the key elements and steps of assessing health system/program performance through effective coverage metrics including kinds of health interventions assessed using this metrics globally, the criteria for selecting those interventions and their need, use and quality estimation approaches and strategies.

## Methods

To better address our research questions, we applied a systematically scoping review, a new searching method, which aims to map the key concepts underpinning a research area and the main sources and types of evidence available rapidly, especially where an area is complex or has not been reviewed comprehensively before [18].

A scoping review is often compared with a systematic review. Contrary to systematic reviews, scoping studies tend to address broader topics where many different study designs might be applicable, are less likely to seek to address very specific research questions nor, consequently, to assess the quality of included studies [19].

The first search was conducted in February 2016. We performed a search of electronic journals and databases including EMBASE, PubMed, Science Direct, ProQuest, Springer, Scopus and Web of Science using single combined keyword of “effective coverage”. We also searched WHO and World Bank Group websites to get grey literature.

Considering that the concept of effective coverage has recently been added to health literature as a metrics to assess health system performance; in order to capture all the efforts have been done in this subject, no restrictions were put on publications. The authors sought to identify original studies related to assessing effective coverage of health interventions or health systems (mainly adopt WHO’s framework of measuring effective coverage). Collection reviews and commentaries were excluded. We also included relevant conference and meeting abstracts and tried to get corresponding full paper in case it was possible. Relevant studies were identified by abstract and full text screening by two reviewers separately (authors 1 and 4). Studies identified through this strategy were accepted until August 2016. We also did a complementary search up to 3 may 2017 in order to get new records. More detailed selection criteria are presented in Table 1.

**Table 1:** Inclusion and exclusion criteria

	Included	Excluded
Publication type	Original articles Conference and meeting abstracts	Collection Reviews and commentaries Technical reports
Date and language	Any date and language	
Study design	Any study design reporting the health service/intervention assessment, monitoring and evaluation which has been done based on WHOs introduced model of effective coverage	
Setting	All routine health care settings	
scale	Cross country, health system, population, individual and intervention level	
Study population	General population	
Effective coverage assessment	Any health service/intervention assessment, monitoring and evaluation has been done based on WHOs introduced model of effective coverage	No quality for primary outcomes Studies applied Tanahashi’s coverage model to estimate the actual coverage and other models Some fields that effective coverage has especial meaning in them like vaccine coverage and malaria and Filariasis control interventions areas
methods	Study reports the methods used to estimate effective coverage by measuring need, use and quality dimensions	
outcome	Measures reported of effective coverage of health services/interventions	Aimed to assess effective coverage but reported crude coverage (no measurement for quality)

## Results

We found 6647 records based on initial and updated search. These records decreased to 926 after title screening and omission of duplications. Six hundred ninety-two records were excluded based on abstract screening and finally 234 records were included for full text analysis. Totally 52 records were related to effective coverage of health system or interventions. Of these, 18 contributed to the findings on monitoring effective coverage of health interventions and were included in the review. The other 34 were excluded based on exclusion criteria which majority of them was because of the followings:

- The use of other frameworks, models and concepts (like Tanahashi model which is mainly used in bottleneck analysis studies to determine bottlenecks in health service provisions; and incorporates coverage according to five measures of availability, accessibility, initial utilization, continuity, and effective coverage) [20].
- Applying formulas and components other than introduced effective coverage calculating formula (for example, using of other especial formulas to calculate the effectiveness of vaccines with the specific components such as input determinants and efficacy, following-up the full vaccination schedule or in malaria coverage cases, concepts like distribution of bed nets, using of insecticides, early treatment of fever).

**Fig. 1:**
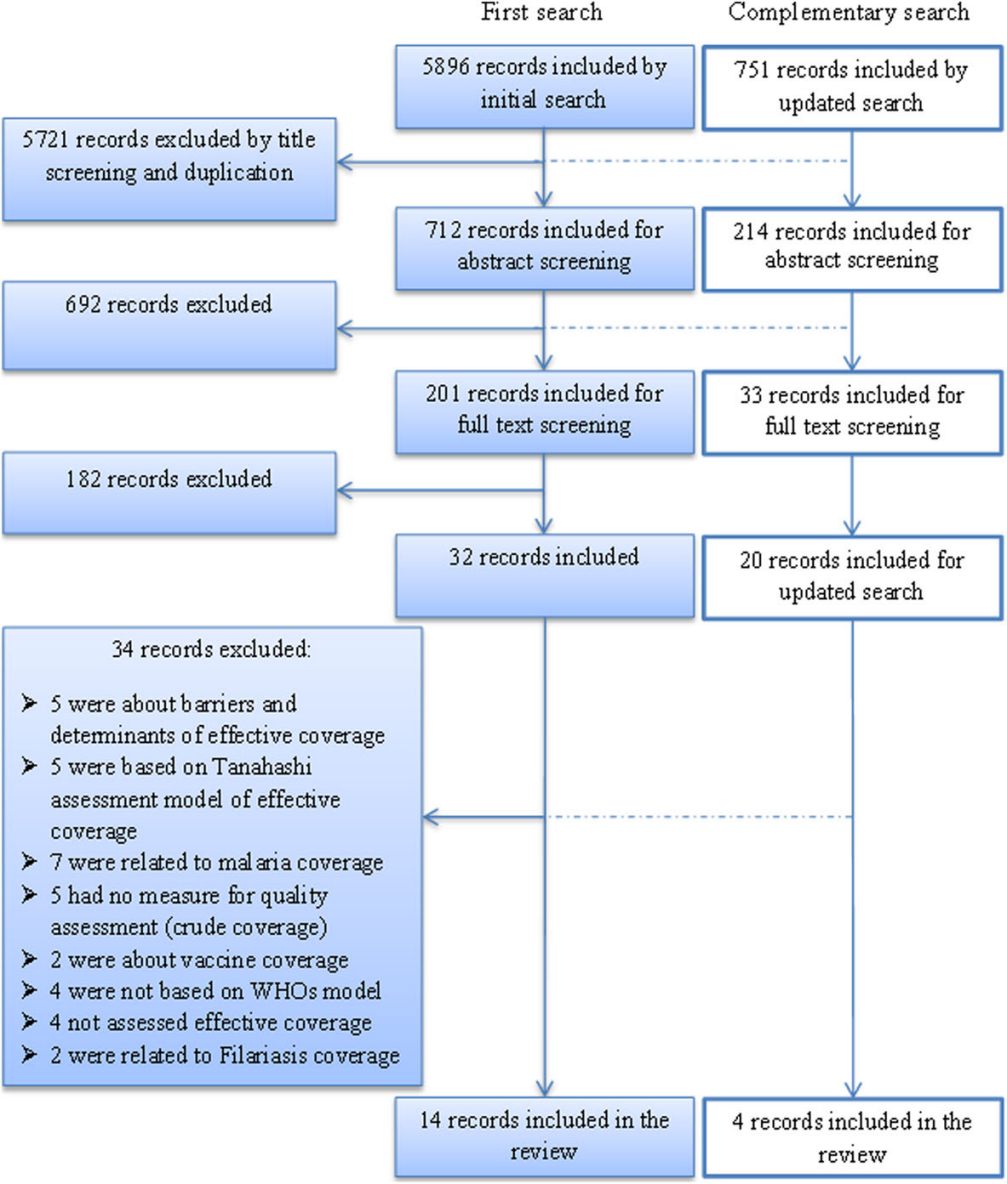
Process of selecting studies

Two of the articles had been published in Spanish and the rest in English (Fig. 1).

Table 2 and Additional file 1: Table S1 provide the indicators and associated measurement strategies for the 18 studies included in the review.

**Table 2:** Interventions monitored by effective coverage with their related components

No	Author	Year	Country	Monitored intervention	Need assessment strategy	Use assessment strategy	Quality assessment strategy	Intervention selection criteria
1	Gakidou E. et al [12]	2006	Mexico	Skilled birth attendance Care of premature neonates Acute respiratory infections treatment in children Hypertension treatment	**National Health Surveys** **Administrated data**	**National Health Surveys** **Administrated data** hospital discharge data	**Biomarkers** **Risk-adjusted outcome** **Content of care** (Not exactly mentioned)	Availability of data for 2000 and 2005–06 comparison
2	Lozano R. et al	2006	Mexico	Antenatal care Skilled birth attendance Services delivered to premature babies Treatment of acute respiratory infections in children Treatment of vision disorders Glycemic control in diabetes Treatment of hypertension Treatment of hypercholesterolemia	**Normative** **Biomarkers** **Self-reported diagnoses** from household surveys	**National** **Health Surveys** Self-reporting	**Biomarkers** **Risk-adjusted outcome** **Content of care**	Projected impact on the burden of disease, affordability, potential impact on health disparities, and ability to extrapolate from these interventions to other interventions Data availability
3	Liu Y. et al. [22]	2008	china	Hypertension treatment	**National Health Survey** self-reporting	**Household Surveys** self-reporting	**Biomarkers** Physical examination included measurement of blood pressure	China’s major health problems Data availability
4	Gakidou E. et al. [29]	2008	57 Countries	Cervical cancer screening	**Normative** eligible women aged 25 to 64	**World Health Surveys** self-reporting	Self-reporting World Health Surveys	Second most common cancer in women and a leading cause of mortality worldwide
5	Idzerda L. et al. [34]	2011	Serbia	Acute respiratory infection treatment	**Normative** children less than five years	**Household Surveys** self-reporting of receiving medication	**Biomarkers** a lab test confirming the presence of ARI before antibiotics are prescribed and again after the treatment course confirming that the ARI has been resolved.	leading cause of death in children under 5 years old in Serbia
6	Martínez S. et al. [11]	2011	Latin American countries	**a) Child Health** Breastfeeding **b) Women’s Health** Prenatal control Childbirth care by qualified personnel Cervical cancer screening Breast cancer screening **c) Adult Health (Chronic Diseases)** Treatment of hypertension Treatment of diabetes Treatment of hypercholesterolemia	**Standard, Single symptom or multiple symptoms, Biomarkers or Performance tests**	**Household surveys** self-reports on care **Administrative data** records of interventions, drug inventory, **Biomarkers**	**Biomarkers, Self-report, Process measures, Rates of mortality**	Availability of data
7	López-López E. et al. [31]	2012	Mexico	Type 2 diabetes actions	**National** **Health Surveys**	**Administrative Data** records of the diabetes control program	records of the diabetes control program	High occurrence and prevalence rate
8	Randive BB et al. [35]	2013	India	Institutional deliveries (caesarean section rate as a proxy)	**Administrative data** District caesarean section rates	**Administrative Data** Population-level estimates of ID and caesarean section proportions	This fraction was multiplied by the ID proportion to give an effective coverage	High maternal mortality ratio
9	Colson KE. et al. [26]	2013	Mexico	Measles immunization	**Normative** children aged 12–23 months	**Household Surveys** self-reporting	**Biomarkers** Seroconversion blood spot samples	
10	Viviescas-Vargas DP. et al. [30]	2013	Mexico	Primary care services for the management of domestic violence against women	**household surveys** based on questionnaire	**Household Survey** self-reporting	household surveys	High prevalence rate
11	Colson KE. et al. [25]	2015	Mexico and Nicaragua	Measles Immunization	**Normative** children aged 12 to 23 months	**Household Survey** caregiver recall **Administrative Data** child health cards	**Biomarkers** dried blood spots (DBS) serological methods	Vulnerability to imported cases and outbreaks in both countries
12	Engle-Stone R. et al	2015	Cameroon	Programs to control vitamin A deficiency	**Normative** women 15–49 years children 6–59 months	**National Health Surveys**	**Biomarkers** Prevalence of inadequate VA intake at “baseline coverage” as the proportion of the population that has inadequate intake at baseline and then “achieves sufficiency” as a result of a given intervention or set of interventions.	Prioritizing and coordinating micronutrient intervention programs Deficiencies in micronutrients contribute to excessive morbidity and mortality of young children globally
13	Travassos MA. et al. [24]	2016	Ethiopia	Immunization tetanus Haemophilus influenzae type b (Hib)	**Normative** children aged 12–23 or 6–8 months	**Administrative Data** vaccination cards and immunization clinic records **household survey** maternal recall	**Biomarkers** Serosurveys	Conflict in coverage rate of different sources
14	Ríos-Blancas MJ. et al	2016	Mexico	Hypertension treatment	The prevalence of hypertension Physical examination in national health survey biomarkers	self-report of antihypertensive treatment	**Statistical methods** two-step least squares method (MC2E) was used using instrumental variables (VI) instrumental variables	One of leading causes of death Burden of Disease
15	Luo H. et al. [27]	2016	Cameroon	programs on adequacy of folate and vitamin B-12 intake	**Normative** Women children 12–59 months	**National Health Surveys**	**Biomarkers** evaluated the baseline prevalence of inadequate intake and biochemical deficiency of these nutrients analysis of blood specimens	High inadequate rates of folate and vitamin B-12 intake
16	Ramke J. et al. [36]	2017	20 countries	Cataract surgery	number of people having operable cataract	number of people with operated cataract	**Biomarker** presenting visual acuity in the operated eye	leading cause of blindness globally
17	Guerrero-Nunez S. et al. [33]	2017	Chile	Diabetes Mellitus Type 2 control	diabetic patients prevalence rate	diabetic patients participating in the health control program	**Biomarker** Percentage of patients in metabolic control [HbA1c < 7%], according to the prevalence of Diabetes Mellitus type 2 at the national and regional level	morbidity and mortality rate guaranteed pathologies
18	Nguhiu PK. et al. [23]	2017	kenya	**Maternal and child health interventions** Family planning services Functional antenatal services Skilled delivery and perinatal care Breastfeeding during the first 6 months of life Immunization services Management of diarrhea Care seeking for acute respiratory illness and/or fever Use of insecticide-treated nets	**Normative**	**National Health Surveys Administrative data**	**Content of care**	The recommendations of the Commission on Information and Accountability for Women and Children’s Health Report based on their relevance to national priorities Availability of data

Bold entries in three strategy categories: main strategies

### Assessment scope

#### Health system assessment by identifying proxy interventions

Only four studies tried to monitor health system performance by selecting proxy interventions (interventions which could be better representative of an area of care or health system) (Table 2). Lozano and others, in 2006, started the first effort in assessment of country health system performance by effective coverage with identifying proxy interventions in both preventive and curative dimensions. They selected 18 interventions opportunistically based on available data but could measure effective coverage for eight of them [21]. Another similar study was carried out in the same country but for limited interventions [21]. Martinez and colleagues conducted a larger study across Latin American countries in 2011 aiming at assessing effective coverage of selected interventions in three areas including infant health, women’s health and adult health [11]. In Liu and others’ work, 11 interventions were selected for China’s health system performance but due to data restriction, they could estimate effective coverage only for 1 intervention [22]. So, we excluded this study from health system assessment category. In 2017, Nguhiu et al. conducted an effort to assess 8 maternal and child health interventions in Kenya [23].

#### Specific interventions assessment

The rest of the studies were carried out in order to use effective coverage for the assessment of specific program(s) or intervention(s). Some of these efforts have been done for preventive interventions such as immunization [24–26], programs to control micronutrients [27, 28], cancer screening [29], and social support for women [30]. Also, there have been attempts to assess some treatment interventions for chronic diseases [22, 31–33], hospital care for women and children [34, 35] and cataract surgery [36].

### Selected interventions areas

Both proxy interventions and specific interventions selected to be monitored by effective coverage mainly were related to the following care areas:

#### Child health

In this category, studies were conducted for both preventive interventions including immunization efforts for complete set of vaccines [23] as well as for specific vaccines including measles [25, 26], tetanus and haemophilus influenza type b vaccinations [24], programs to control vitamin A [28] and folate and vitamin B-12 [27] deficiency in children, breastfeeding [11, 23], and treatment interventions including acute respiratory infections in children [21, 23, 34], management of diarrhea [23] and care of premature neonates [21].

#### Prenatal and antenatal care and delivery

Interventions related to prenatal and antenatal care and delivery such as prenatal control, childbirth care by qualified personnel, and institutional deliveries [11, 21, 23, 35] have been opted as a proxy in all of health system assessment studies and as a specific subject for monitoring.

#### Chronic conditions

Some of the studies tried to assess effective coverage of chronic conditions and diseases in both preventive and curative areas. Prevention and treatment of hypertension [11, 21, 22, 32], prevention and treatment of diabetes [11, 21, 31, 33], treatment of hypercholesterolemia [11, 21], treatment of vision disorders [21, 36], breast [11] and cervical cancer screening [11, 29] were among prioritized interventions selected to be monitored.

### Intervention selection criteria

There has been logic for the selection of interventions for monitoring their effective coverage, especially for the proxy ones. Potential impact on the burden of disease and health disparities, affordability, and ability to extrapolate from the selected interventions to other interventions have been mentioned as main selection criteria in Lozano and others [21, 37]. Although Martinez and his colleagues have not explicitly mentioned intervention selection criteria in their work, an appropriate combination of interventions in various fields condition on the comparability of data between the countries under study was a concern [11]. This is true for Gakidou et al. study too, except that they considered data availability between two periods of comparison in the same country [21]. Selection of maternal and child interventions in Nguhiu et al. study was based on the recommendations of national authorities because of their relevance to national priorities [23].

Country’s major health problems and high-priority conditions [22], Burden of Disease and leading causes of mortality and morbidity [29, 32–34], high maternal mortality ratio [35], high occurrence and prevalence rate [30, 31, 36] and conflict in coverage rate of different sources [24] were among the main selection criteria in specific interventions assessment category. In addition to the mentioned criteria, availability of data was the critical prerequisite especially in all of the studies applied *ex post* approach in estimating effective coverage.

### Strategies to estimate need, utilization and quality components of effective coverage

#### Need assessment strategies

In almost half of the studies according to the content of the selected interventions, *need* has been defined and measured normatively. Based on demographic data, individuals who needed the interventions related to particular age [23–28, 34] or sex [11, 23, 27–29] groups were defined and measured based on the defined scope of study. These studies mainly were related to the maternal and child area. Some of the studies benefited from *national health surveys* and *administrative data* [15, 21, 30, 35, 36] in need measurement. In addition to the aforementioned methods, estimating *prevalence rate* through available data on biomarkers or performance tests from health surveys have been used for treatment interventions such as treatment of hypertension, diabetes and hypercholesterolemia in order to measure the denominator [11, 21, 32, 33].

#### Utilization assessment strategies

Most of the studies have used available *self-reporting data from household surveys* [11, 24–26, 30, 32, 34] and *national health surveys* [21, 23, 27–29, 34] in order to estimate the *utilization*. Another method used in this regard was *administrative data* [11, 21, 23–26, 31, 33, 35] such as program-specific control records for diabetes, hospital discharge data, and drug inventory.

#### Quality assessment strategies

Based on our findings, the methods and strategies used to measure the quality component of effective coverage (interventions effectiveness) varied among studies based on the type of selected interventions. *Biomarker* measurement has been used in the majority of studies including immunization efforts [25, 26, 30], programs to control micronutrients [27, 28] control of chronic conditions [33] and surgery [36]. Also, some health system assessment efforts benefited from biomarkers and physical examinations acquired from periodic national health surveys as a part of combined methods for measuring intervention effectiveness [11, 21]. Nguhiu and others used *content of care* strategy in assessing maternal and child interventions [23]. This method can be found in Lozano et al. study too [21]. *Statistical methods including instrumental variables* was the other method applied to assess hypertension treatment assessment [32]. *Risk-adjusted outcome* (mortality) was part of a mixed strategy which was used to assess services delivered to premature babies in Lozano et al. study [21].

## Discussion

The first step in implementing effective coverage as a performance assessment tool is selection of interventions. Ideally, WHO suggests using effective coverage as a measure of the performance of national health system as a whole [9]; however, it can be measured for a specific health intervention individually, [17]. For the former, main interventions as health system representatives should be identified [12] and then, effective coverage of these set of interventions can be aggregated into the effective coverage of the health system [7]. The representatives should be chosen in such a way that can indicate coverage level of the other interventions with similar content [38].

There have been experiences for both of these assessment scopes (health system or specific program) in Mexican health system. Totally, the number of health systems tried to use and develop effective coverage concept to assess their health system performance is a handful. For the first time, effective coverage was measured for some proxy public health interventions in Mexico [21]. Although there have been some criticisms on the selected interventions as well as measurement effective coverage for all of these interventions and for Mexican health system as a whole [39], it is worthwhile as the first attempt in developing practical concepts of effective coverage and a performance benchmarking device among Mexican states.

But, which interventions should be selected to evaluate and what criteria should be considered for the selection? Given the wide range of health interventions delivered by health systems, measuring effective coverage for all of them would be impossible [15, 37]. In health system assessment efforts, priority interventions which could both address the most important health needs of population and be a good set of proxies at all levels should be considered [7, 40, 41]. It is obvious that the optimal set of interventions vary across countries or even districts as a function of national and local epidemiology and other characteristics. In the studies related to health system assessment [11, 21, 23], there has been an attempt to capture interventions that can cover the main above mentioned levels. Especially, Martinez and colleagues’ work has a proper category of interventions, which covers child health, women’s health, and adult health (chronic diseases) areas [11].

When selecting which interventions to include in estimating effective coverage, some considerations should factor such as burden of disease, affordable interventions, and special considerations of social priority [1]. For this, existing evidence on health need priorities should be examined, cost-effectiveness of interventions should be evaluated, and political, social, and cultural concerns of equity should be considered [37]. What is important here is that the availability of data has a strong effect on the selection of intervention strategies, especially in ex post approaches to measurement. To measure the effective coverage of health interventions in China, Liu and others selected those interventions that were relevant to China’s major health problems but because of data limitation, they could only estimate effective coverage for one of them [26]. To better deal with this issue, applying ex ante methods is suggested in designing assessment projects [10].

Monitoring effective coverage of health system in countries experiencing epidemiological transition not only should be designed to capture the main interventions that address non-communicable diseases, such as hypertension and diabetes control and most common mental disorders that estimated effective coverage for them are on average about half the coverage rates not adjusted for the success rate of treatment [22, 41, 42], but also the proposed framework should include the set of interventions related to the health MDGs, with a focus on communicable diseases and maternal and child health interventions, especially in health systems where these conditions are still a priority [7, 12].

According to the findings, measuring effective coverage of maternal and child health interventions has been included in both health system assessment and specific intervention assessment efforts. Their ability to produce a significant health gain in a relatively short time, their correspondence to the priorities and objective needs of the countries, existing ample evidence for their effectiveness and a response to a significant health problem at national and regional level made maternal and child health interventions proposed by the WHO 2001 technical consultation as high priority areas for selecting interventions for effective coverage assessment [5]. Also, there is relatively little additional cost to obtain the data for calculating effective coverage for maternal and child health interventions [7].

None of studies related to health system assessment could estimate effective coverage for immunization interventions because of their ex post approaches. Three studies were conducted aiming at estimating vaccination effective coverage by defining ex ante approaches and strategies to capture quality component of coverage [24–26]. This area is a good example to compare crude and effective coverage of interventions and represent the gap between utilization of services and what is actually delivered.

As mentioned earlier, to estimate effective coverage of interventions, the metric’s three components— intervention need, use, and quality — need to be measured in a consistent way [37]. For this purpose, approaches and strategies must be accurately determined. Clear definitions of the content of an intervention, therefore, are essential for any realistic measurement approach.

How the denominator and numerator should be estimated? People in need are not simply those who demand a service, but a true population measure of those who would benefit from receiving a specific health intervention [21, 43]. Need can be defined normatively; for example, an entire group of individuals such as all children at certain ages or all pregnant women. For many treatment interventions such as antiretrovirals, or hypoglycemic agents for diabetes, however, need is much more challenging to assess [1]. Both of these strategies were applied in health system assessment and specific intervention assessment studies (Table 2).

The use of an intervention is a central component of measuring effective coverage [37]. Data systems for measuring the number of people receiving an intervention mainly are self-reporting data from household surveys and administrative data [1].

Due to the high probability of biases in information systems, especially in weak health systems, properly measuring intervention use and tracking intervention coverage over time can be challenging, especially if there would be more emphasis on administrative data [37]. To deal with this problem, other complementary strategies are used like household surveys as a key source of coverage information [26].

In order to achieve a more accurate measurement of need and use, therefore, advantages and disadvantages of each method should be considered according to the content of interventions and tried to, as much as possible, take a combination approach. It is clear that in health system assessment efforts, applying combination of strategies and methods is inevitable. Lozano et al. and Martinez et al. are good examples in this regard [11, 21]. Because of applying ex post approach in most of studies, there has been more reliance on existing data from surveys rather than designing new strategies.

As previously mentioned, in effective coverage approach, quality means the potential health gain of delivered interventions. Due to the variety and the nature of interventions in the health system, health outcomes cannot be measured by using a single method. For example, an indicator of assessing the effectiveness of “AIDS treatment” will vary with the “prevention of overweight or obesity” one. For the first one, we may need CD4 counting test, while for the latter, measurement of the body mass index would be sufficient. Measuring quality–fraction of potential health gain that is actually delivered is the most important and at the same time the most challenging area of measuring effective coverage and the one for which the most development is needed [43]. This is why most of the efforts in measuring effective coverage have failed and only relied on the measurement of the crude coverage. Although assessing intervention quality is often the most complicated aspect of estimating effective coverage, a variety of approaches and strategies have been developed to measure the quality of interventions by the pioneers of this concept. Some of these main strategies are content of care, biomarkers of effectiveness, cohort registration, exposure matching in household survey data, statistical methods including instrumental variables, case–control methods and risk-adjusted outcomes [1, 10, 37].

In most of the studies concerning effective coverage, the analysis was restricted to routinely collected data that was already available (from health and nutrition surveys etc.). In these studies, therefore, quality had to be measured *ex post* either based on the data available, which may be inadequate indicators of health gain. According to the findings, health system assessment studies were unable to find data on the quality of immunization and some treatment interventions and; therefore, could only report the crude coverage of them (of course, these cases have been excluded from the findings because of non-calculation of the quality component). In ex ante approach to measure quality, there is the opportunity to define the indicators of quality beforehand and collect data adequate for the analysis of effective coverage, thereby, contribute new knowledge, data and methodologies to the field of health system assessment [7].

### Capacity building to measure effective coverage of health systems

Along with increasing capacity of health systems, more emphasis should be undertaken on shifting performance assessment approaches from paying attention to indicators on coverage (availability, access or use) to determine the quality and actual health outcomes of the interventions as well as effective coverage, which goes far beyond just access [7, 41, 44, 45]. With the emergence of new initiatives in the realm of international health agenda such as universal health coverage and commitment of local health systems to the pursuit of their goals, strengthening health systems capacity to monitor population health, effective coverage of interventions, health risks, and health outcomes through designing new performance assessment frameworks, approaches, methods and tools is necessary in order to comply with these initiatives [12, 34, 44, 46]. Mexico is a good example of a country that has taken a step in this direction and tried to operationalize newly recommended concepts.

Usually, in weak health systems in low-income settings, because of less developed data systems and poor capacity of primary and secondary data, which are critical to measure effective coverage [1], it is not easy to maneuver in the area of performance assessment. In these contexts, with considering limitations of measuring effective coverage [47], more efforts should focused on periodical small size assessment programs such as district level and state-level through a combination of survey and research efforts across many disciplines in implementation of science rather than national wide evaluations [15, 44, 48]. On the basis of the assessment, gaps and unmet monitoring and evaluation needs in national surveillance systems can be identified and strategies can be developed to address those needs [49].

## Conclusions

The gradual theoretical development of effective coverage concept and increasing practical efforts along with it in recent years illustrate the potential of this approach and its applicability as a practical metrics of health system performance assessment. However, future studies are required to contribute to the ongoing improvement, especially through defining prospective approaches and strategies into estimation of composite measures based on the effective coverage framework. Also, further attention needs to be paid to quality measures of effective coverage in a manner that better linkage would be established between coverage rates and interventions effectiveness. In addition, at the administrative system level, more innovation and further improvements are needed to develop the existing surveillance systems in order to enhance capacity of routine health service statistics, demographic and epidemiological data for the essential health interventions, especially in developing countries. As the last word, it should be considered that constant and expanded assessment of effective coverage of health interventions, whether preventive, curative or palliative across countries over time is an important output of health systems that would pave the progress way toward Universal Health Coverage.

## Additional file

Additional file 1: **Table S1.** Interventions monitored by effective coverage in health system assessment efforts and their detailed components. (DOCX 36 kb)

## Abbreviations

EC: Effective coverage
UHC: Universal Health Coverage
WHO: World Health Organization

## Footnotes

1 The concept of quality as defined in effective coverage approach has much in common with that of effectiveness, however, in order to maintain consistency with the literature on effective coverage, the word quality will be used for the purpose of this paper.
