# Supplementary material for: Effective coverage as a new approach to health system performance assessment: a scoping review
Source: BMC Health Serv Res. 2018 Nov 23;18:886. doi: 10.1186/s12913-018-3692-7 (PMC6251131; doi:10.1186/s12913-018-3692-7)
Supplement: Supplementary file 1 — Table S1. Interventions monitored by effective coverage in health system assessment efforts and their detailed components. (DOCX 36 kb) [file 12913_2018_3692_MOESM1_ESM.docx]

**S1 Table. Interventions monitored by effective coverage in health system assessment efforts and their detailed components**

| **No** | **Author** | **Year** | **Country** | **Monitored intervention** | **Need assessment**  **strategy** | **Use assessment strategy** | **Quality assessment strategy** | **Intervention selection criteria** |
| --- | --- | --- | --- | --- | --- | --- | --- | --- |
| 1 | Gakidou E. et al | 2006 | Mexico | Skilled birth attendance  Care of premature neonates  Acute respiratory infections treatment in children  Hypertension treatment | **National Health Surveys**  **Administrated data** | **National Health Surveys**  **Administrated data**  hospital discharge data | **Biomarkers**  **Risk-adjusted outcome**  **Content of care**  (Not exactly mentioned) | Availability of data for 2000 and 2005–06 comparison |
| 2 | Lozano R. et al | 2006 | Mexico | Antenatal care | **Normative**  **Biomarkers**  **Self-reported diagnoses**  from household surveys  Women who gave birth in a given time  period | **National**  **Health Surveys**  Self-reporting  Saw a doctor, nurse, or midwife during  pregnancy at least four times | **Biomarkers**  **Risk-adjusted outcome**  **Content of care**  Received blood test and had blood pressure measured | Projected impact on the burden  of disease, affordability, potential impact on health disparities,  and ability to extrapolate from these interventions to other interventions  Data availability |
|  |  |  |  | Skilled birth attendance | Women who gave birth in a given time  period | Birth attended by health professional | Birth took place in hospital |  |
|  |  |  |  | Services delivered to premature babies | Babies born at 28–36 weeks gestation  or those weighing <3500 g at birth | Birth took place in hospital | Difference in mortality rate in premature babies compared with maximum and minimum risk-adjusted mortality |  |
|  |  |  |  | Treatment of acute respiratory infections in children | Children younger than 5 years whose  mothers report their having had a cough, cold, flu, bronchitis, or earache in the 2 weeks preceding the survey | Mother reports child received treatment | Treatment from a health worker |  |
| **No** | **Author** | **Year** | **Country** | **Monitored intervention** | **Need assessment**  **strategy** | **Use assessment strategy** | **Quality assessment strategy** | **Intervention selection criteria** |
|  |  |  |  | Treatment of vision disorders | Adults older than 20 years who report  near or far visual impairment or wear  glasses or contact lenses | Use glasses or contact lenses | Report no near or far visual impairment when wearing glasses or contact lenses |  |
|  |  |  |  | Glycemic control in diabetes | Fasting plasma glucose estimated from casual plasma glucose ≥6.9 mmol/L | Self-reported use of oral hypoglycemic  or insulin | Reduction in fasting plasma glucose compared with treatment targets |  |
|  |  |  |  | Treatment of hypertension | Adults older than 20 years with systolic  blood pressure ≥140 mm Hg | Self-reported use of antihypertensive  agents | Reduction in systolic blood pressure compared with treatment targets |  |
|  |  |  |  | Treatment of  hypercholesterolemia | Adults older than 20 years with total  cholesterol ≥5.2 mmol/L | Self-reported use of drugs for cholesterol  reduction | Reduction in total cholesterol compared  with treatment targets |  |
| 3 | Martínez S.  et al | 2011 | Latin American countries | Maternal and child health interventions:  **a) Child Health**  Breastfeeding | **Standard, Single symptom or multiple symptoms, Biomarkers or Performance tests**  Children under 6 months | **Household surveys** self-reports on care  **Administrative data**  records of interventions,  drug inventory, **Biomarkers** | **Biomarkers,** self-report, process measures, rates of mortality  Measure in terms of development of acute diarrheal disease (EDA) and acute respiratory infection (ARI). | Availability of data |
|  |  |  |  | **b) Women's Health**  prenatal control | Women who during the last 5 years were pregnant (last pregnancy). | Women who during their last pregnancy Attended at least four times to prenatal quality control. | Measured in terms low birth weight |  |
| **No** | **Author** | **Year** | **Country** | **Monitored intervention** | **Need assessment**  **strategy** | **Use assessment strategy** | **Quality assessment strategy** | **Intervention selection criteria** |
|  |  |  |  | Childbirth care by qualified personnel | Women who gave birth in the last five years (last childbirth). | Women who during the last childbirth (last five years) were attended by qualified personnel. | Measured in terms of complications during childbirth or maternal mortality. |  |
|  |  |  |  | Cervical cancer screening | Women between 15 and 49 years old. (The following age groups were differentiated for this indicator: women less than 25 years of age, between 25 and 49 years and over 50 years). | Women aged 15-49 who underwent cytology during the past year. | Measured in terms of the proportion of women who have had abnormal results and have been treated. |  |
|  |  |  |  | Breast cancer screening | Women between 40 and 49 years old | Women between 40 and 49 years old who had mammograms in the last year. | Measured in terms of women who have had abnormal results and who have been treated. |  |
|  |  |  |  | **c) Adult Health (Chronic Diseases)**  Treatment of hypertension | People between 18 and 69 years old diagnosed with hypertension. | People diagnosed with hypertension who are under pharmacological treatment. | Measured in terms of patients diagnosed with hypertension who are under treatment and who are controlled. |  |
|  |  |  |  | Treatment of diabetes | People between 18 and 69 years old diagnosed with diabetes. | People diagnosed with diabetes who are under pharmacological treatment. | Measured in terms of patients diagnosed with diabetes who are under treatment and who are controlled. |  |
|  |  |  |  | Treatment of hypercholesterolemia | Persons between 18 and 69 years old diagnosed with hypercholesterolemia. | Persons with cholesterol levels above 200 mg /dl who are being treated. | In terms of diagnosed patients who are controlled. |  |
| **No** | **Author** | **Year** | **Country** | **Monitored intervention** | **Need assessment**  **strategy** | **Use assessment strategy** | **Quality assessment strategy** | **Intervention selection criteria** |
| 4 | Nguhiu PK. et al | 2017 | kenya | **maternal and child health interventions**  Family planning services | **Normative**  Women 15–49 years old who at the time of survey were able to get pregnant | **National**  **Health Surveys**  **Administrative data**  Fecund women 15–49 years old,  currently using a modern contraceptive method | **Content of care**  Facility level score based on the presence of client privacy during consultation, availability of reproductive health counseling visual aids and record tools, and reproductive health commodity management practices in a facility. | The recommendations of the Commission on Information and  Accountability for Women and Children’s Health Report based on their relevance to national priorities  Availability of data |
|  |  |  |  | Functional antenatal services | Women 15–49 years old with at least one child under 5 years | Women 15–49 years old with at least one child under 5 years, whom for their most recent birth, reported  having made at least four visits for  ANC | Individual level score if the respondent recalls the following services being performed: blood pressure taken, urine sample taken, blood sample taken, respondent informed about pregnancy complications, iron tablets/syrup prescribed, and a drug for intestinal parasites prescribed, during any ANC visit. |  |
|  |  |  |  | Skilled delivery  and perinatal  care | Women 15–49 years old with at least one child under 5 years | Women 15–49 years old with at least one child under 5 years,  whom for their most recent birth, reported attendance by a skilled health provider (doctor, nurse or midwife) | Facility level score based on reported routinely performed essential newborn care practices at the facility maternity. These included routine rooming in with the mother, routine weighing of newborns, complete examination of newborns before discharge, administration of BCG before discharge and other indicators. |  |
| **No** | **Author** | **Year** | **Country** | **Monitored intervention** | **Need assessment**  **strategy** | **Use assessment strategy** | **Quality assessment strategy** | **Intervention selection criteria** |
|  |  |  |  | Breastfeeding  during the first 6 months of life | All children between 0 and 5 months | All children between 0 and 5 months, for whom breastfeeding was reported in the preceding 24 h | Individual level: All children  between 0 and 5 months for  whom exclusive breastfeeding (breastfeeding only, with no other complementary feed offered) was reported in the preceding 24 h |  |
|  |  |  |  | Immunization  Services | All children alive between 12 and 23 months | All children alive between 12 and 23  months who received the complete set of vaccines as outlines in the Kenya Ministry of Health National  Vaccination Schedule i.e. BCG, three doses of oral or intravenous Polio, three doses of Diphtheria, Pertussis,  Tetanus, Hepatitis B and Hemophilus Influenza type B  pentavalent vaccine, three doses of pneumococcal vaccine (from  Jan 2011 onwards), and Measles vaccines | Facility level score based on  observed or health worker  reported availability of at  least one working weighing  scale and thermometer, and routinely performed processes including use of guidelines to assess and treat sick children, routine weighing, temperature taking and recording, assessment of immunization status and keeping of individual patient records. |  |
|  |  |  |  | Management  of diarrhea | All children under 5 years reported to have had diarrhea in the preceding 4 weeks | All children that had diarrhoea in the preceding 4 weeks, who were given oral rehydration therapy  (ORT) or increased fluids. | Individual level: Proportion of children who had diarrhoea in the preceding 4 weeks, who were given the guideline recommended oral  rehydration salt mixture |  |
| **No** | **Author** | **Year** | **Country** | **Monitored intervention** | **Need assessment**  **strategy** | **Use assessment strategy** | **Quality assessment strategy** | **Intervention selection criteria** |
|  |  |  |  | Care seeking  for acute respiratory  illness and/or fever | All children under 5 years reported to have had acute respiratory illness and/or fever  in the preceding 2 weeks | All children who had acute respiratory  illness and/or fever, for whom advice  on treatment was sought from a  medical provider | Facility-level score based on observed or health worker reported availability of at least one working weighing scale and thermometer,  and routinely performed processes including use of guidelines to assess and treat sick children, routine weighing, temperature taking and recording, assessment of immunization status and keeping of individual patient records |  |
|  |  |  |  | Use of insecticide  -treated nets | All children and pregnant women | Proportion of children and pregnant  women living in household that owned an ITN | Individual level: Proportion of children and pregnant women who actually slept under an insecticide treated  net in the preceding night. |  |
